# Supplementary material for: Safety and Quality of Canned Sardines after Opening: A Shelf-Stability Study
Source: Foods. 2022 Mar 29;11(7):991. doi: 10.3390/foods11070991 (PMC8997536; doi:10.3390/foods11070991)
Supplement: Supplementary file 1 [file foods-11-00991-s001.zip › foods-1649118-supplementary.pdf]

Safety and quality of canned sardine after opening: a shelf-stability study

Rebeca Cruz[1*], Vânia Pereira[1], Teresa Pinho[1], Isabel Ferreira[1], Carla Novais[2] and Susana Casal[1,3,*]

[1]LAQV/REQUIMTE, Laboratório de Bromatologia e Hidrologia, Faculdade de Farmácia, Universidade do Porto, Rua de Jorge Viterbo, Ferreira 228, 4050-313, Porto, Portugal.

[2]UCIBIO, Laboratório de Microbiologia, Faculdade de Farmácia, Universidade do Porto, Rua de Jorge Viterbo Ferreira 228, 4050-313, Porto, Portugal.

[3]EPIUnit e Instituto de Saúde Pública, Universidade do Porto, Rua das Taipas 135, 4050-600, Porto, Portugal.

*Corresponding author: rcruz@ff.up.pt

Table S1. Relative composition and labelled shelf life of canned sardines.

ID	Ingredients		Net weight/Drained weight (g/g)	Shelf life (years)
	Description	Percentage (%)		
**Sardine in light brine (SB)**	Sardine	70	120/84	3
	Water	29		
	Salt	1		
**Sardine in vegetable oil (SO)**	Sardine	70	120/84	5
	Vegetable oil	29		
	Salt	1		
**Sardine in tomato sauce (ST)**	Sardine	65	120/78	4
	Tomato	30		
	Vegetable oil	4		
	Salt	1		
